# Prediction and risk factors for one year mortality in patients after surgery for pyogenic spondylodiscitis

**DOI:** 10.1007/s10143-025-04114-8

**Published:** 2026-01-24

**Authors:** B. Younes, B. Schatlo, D. Mielke, V. Rohde, T. Abboud

**Affiliations:** 1 Department of Neurosurgery, University Medical Center Göttingen, Robert-Koch-Straße 40, 37075 Göttingen, Germany; 2 Department of Neurosurgery, University Hospital Augsburg, Augsburg, Germany

**Keywords:** Pyogenic spondylodiscitis, One year mortality, Mortality, Prediction and risk factors

## Abstract

**Objective:**

Pyogenic spondylodiscitis is a severe spinal infection often requiring surgical intervention, with notable one-year mortality rates. This study aimed to identify factors associated with 1-year mortality following instrumented surgery to guide postoperative care and surgical planning.

**Methods:**

A retrospective analysis was conducted on 370 patients who underwent instrumented surgery for pyogenic spondylodiscitis (2013–2022). Univariate and multivariate logistic regression analyzed factors associated with 1-year-mortality, including age, Charlson Comorbidity Index (CCI), neurological deficits, C-reactive protein levels (CRP), type of surgery, number of spinal levels treated, complications, duration of surgery, and revision surgery. ROC analysis determined optimal cutoff values for significant factors associated with mortality.

**Results:**

Out of the 370 patients included in the study, 38 (10.2%) died within one year of treatment, including 6 (1.6%) within the first 30 days. The mean follow-up period was 24 ± 3 months. Univariate analysis identified the presence of neurological deficits (OR 2.82, 95% CI 1.21–6.55, *p* = 0.0158), last CRP levels (OR 1.009, 95% CI 1.005–1.014, *p* < 0.001), and the number of spinal levels treated (OR 1.35, 95% CI 1.03–1.77, *p* = 0.0294) as significant factors associated with 1-year mortality. Multivariate logistic regression confirmed last CRP (OR 1.01, 95% CI 1.004–1.016, *p* = 0.001) and number of spinal levels treated (OR 1.345, 95% CI 1.006–1.799, *p* = 0.046) as independent factors associated with mortality within one year. Receiver operating characteristic (ROC) analysis identified optimal thresholds for predicting mortality: last CRP > 47 mg/L (AUC 0.767) and more than two spinal levels treated (AUC 0.638).

**Conclusion:**

Elevated CRP levels(> 47 mg/L) at a median of 4 months postoperatively [IQR: 2–6], and extensive spinal surgery involving more than two levels are independent factors associated with one-year mortality. These findings highlight the importance of long-term postoperative CRP monitoring and careful surgical planning to improve outcomes in patients with pyogenic spondylodiscitis.

## Introduction

Between 2005 and 2021, the incidence of pyogenic spondylodiscitis in Germany increased by 104%, rising from 5.4 to 11.0 cases per 100,000 individuals [11]. A comparable trend was observed in England, where the incidence increased by 44% between 2012 and 2021 [24]. These increases are likely attributable to advancements in diagnostic imaging, particularly magnetic resonance imaging (MRI) and the growing proportion of elderly individuals within the population [12, 29]. Accurate identification of the causative pathogen, primarily through blood cultures, is critical for directing effective antibiotic therapy [7, 13]. The standard treatment protocol typically involves the prompt initiation of broad spectrum antibiotics, followed by a tailored regimen based on pathogen identification [4, 5]. Surgical intervention is reserved for patients who do not respond to medical management or who exhibit neurological deficits, severe radicular pain, or spinal instability [17, 22]. But the early surgical intervention significantly reduced the mortality rate within the first six weeks and shortened hospital stays [19]. Despite significant advancements in both antimicrobial and surgical therapies, pyogenic spondylodiscitis continues to pose a considerable risk of morbidity and mortality.

Many studies have demonstrated a significant difference in mortality rates between patients with pyogenic spondylodiscitis treated conservatively and those who underwent early surgical intervention. Conservative management was associated with a 1-year-mortality rate ranging from 13% to 24.2%, compared to 4.2% to 8% for patients receiving early surgery [29]. Similarly, in-hospital mortality rates showed a stark difference, with 18% in the conservative cohort versus 6% in the surgical group [6, 15, 19, 26, 28]. However, some studies on pyogenic spondylodiscitis mortality are limited by heterogeneous cohorts, small sample sizes, or a narrow focus on either medical or surgical factors. Crucially, the interplay between patient specific comorbidities, pathogen profiles (including antibiotic-resistant strains), and surgical variables remains poorly explored. This study leverages a large, homogeneous cohort of surgically treated pyogenic spondylodiscitis patients to address these gaps. It aims to identify factors associated with mortality.

## Materials and methods

### Patients

This retrospective study included patients treated for pyogenic spondylodiscitis at the University Hospital Göttingen from 2013 to 2022. Only individuals who underwent surgical intervention and antibiotic therapy were included, while those managed conservatively or had insufficient documentation were excluded.

### Preoperative course

Preoperative assessments included routine blood tests and blood cultures. All patients received empiric intravenous antibiotics before surgery. Computed tomography (CT) and magnetic resonance (MR) scans were performed to evaluate the presence of spondylodiscitis, bone destruction, spinal epidural abscess, and any spinal deformities.

### Surgical treatment

Percutaneous navigated or robot assisted dorsal pedicle screw instrumentation was the standard treatment. If neither of these techniques was available, screws were placed free hand under X-ray guidance. The underlying surgical principle was to instrument all preoperatively identified infected levels. No major changes to treatment protocols occurred during the study period. Patients with neurological deficits additionally underwent spinal decompression via microsurgical interlaminar fenestration, hemilaminectomy, or laminectomy. In selected cases, a two stage surgical approach was employed, beginning with dorsal transpedicular instrumentation followed by corpectomy, radical debridement, and placement of distractable vertebral body substitutes (Obelisc™, ulrich medical; adjustable from 0° to 15°).

### Postoperative management

Postoperative assessments included a CT scan to evaluate implant positioning, spinal alignment, and the adequacy of any bony decompression. Patients received a two week course of intravenous antibiotics, tailored to microbial culture results or administered empirically if cultures were negative. This regimen was followed by four to six weeks of oral antibiotics, extended to a total of 12 weeks in more complex cases, until CRP and leukocyte levels returned to normal. A hard brace was prescribed for 12 weeks after surgery, after which patients were allowed unrestricted movement.

### Follow up assessments

According to the department’s standard procedure, routine clinical and radiological follow-up, including MRI, took place four weeks after surgery. CRP and leukocyte levels were monitored weekly. Additional clinical assessments and a CT scan were performed at three months and again at one year postoperatively. In cases where CT findings suggested screws loosening or new deformities, follow-up visits were conducted more frequently and for a longer duration.

### Assessment of mortality

Mortality was defined as death from any cause occurring within the first year after surgery. The primary outcome of all-cause mortality within one year postoperatively was confirmed through a review of internal hospital records and electronic patient files, documenting both in-hospital deaths and vital status from follow-up clinical visits. Patients who lacked clinical follow up data at the one year mark were excluded from this analysis. A sensitivity analysis was performed to evaluate the potential impact of patients lost to follow-up. We assessed two extreme scenarios: assuming all patients with incomplete follow-up (*n* = 15) either (1) died within one year, or (2) survived the first year postoperatively. Multivariate logistic regression was repeated under both conditions. Patient-related factors were age, sex, Charlson comorbidity index (CCI), drug abuse, smoking, obesity, osteoporosis and Methicillin-Resistant Staphylococcus aureus (MRSA) pathogen. Pyogenic spondylodiscitis related factors included lumbar, thoracic and cervical involvement, preoperative pain, neurological deficit and the presence of a spinal epidural abscess, and CRP levels at admission. Treatment related factors were antibiotic therapy use prior to surgery, number of operated levels in the lumbar, thoracic and cervical spine, spinal canal decompression, durotomy, surgical technique (dorsal vs. 360° fusion), new deficit, postoperative complications, wound infection, CRP levels at discharge and at follow up, relapse infection and screw loosening.

### Variable definitions

- Neurological Deficit: Defined as any objectively documented motor and/or sensory deficit and/or bowel/bladder dysfunction documented on clinical examination at the time of admission.
- Postoperative Complications: A comprehensive list including both local and systemic complications. This encompassed surgical site infection, implant malposition or failure requiring intervention, new or worsening neurological motor deficit, deep vein thrombosis, pulmonary embolism, myocardial infarction, and acute renal failure.
- Relapse of Infection: Defined as the recurrence of clinical symptoms (e.g., back pain, fever) suggestive of pyogenic spondylodiscitis, accompanied by positive imaging findings (MRI or CT) and/or elevated biochemical markers (CRP, leukocytes), after a period of initial clinical improvement.
- Screw Loosening/Implant Failure: Defined by both radiological evidence (e.g., a radiolucent ‘halo sign’ >1 mm around the screw on CT, screw breakage, or significant implant displacement) and corresponding clinical symptoms (e.g., new or worsening mechanical pain, deformity).
- CRP Measurement Timing:
- Preoperative CRP: Measured within 24 h before the surgical procedure.
- Discharge CRP: The final measurement taken within 48 h prior to hospital discharge.
- Last CRP: The final available measurement obtained based on clinical indication, not routine practice.

### Statistical analysis

Statistical analysis included chi-square tests and univariate logistic regression to identify variables associated with one-year mortality. Missing data were handled using complete case analysis. No imputation methods were employed. The final model included only patients with non-missing values for all covariates. Variables with a p-value < 0.1 in univariate analysis, were included in a multivariate logistic regression model. Variance Inflation Factor (VIF) was calculated to evaluate multicollinearity between predictors. Receiver Operating Characteristic (ROC) curve analysis was used to evaluate the discriminatory power of significant predictors and determine optimal cutoff values. The area under the curve (AUC) was reported with 95% confidence intervals. Missing data were handled using complete case analysis without imputation. All data were collected in Microsoft Excel and analyzed using IBM SPSS Statistics Version 27.0 (IBM Corp., Armonk, NY, USA) and Python (version 3.10) for additional regression and ROC analyses. Statistical significance was defined as a p-value < 0.05.

As a sensitivity analysis, we additionally fitted a time-to-event Cox proportional hazards model with CRP as a time-updated covariate. For each patient, follow-up time was measured from the date of surgery to either death or 1 year, whichever came first. CRP was modeled as a piecewise-constant time-varying covariate using measurements obtained preoperatively, 3–7 days postoperatively, at hospital discharge, and at the last documented follow-up CRP. Interval boundaries were defined at the approximate times of these measurements (day 0, day 5, length of stay in days, and the calendar date of the last CRP measurement relative to the surgery date). The hazard of death within one year was then modeled as a function of time-updated CRP, with and without adjustment for age and the total number of operated levels.

### Ethics statement

This retrospective chart review study involving human participants was in accordance with the ethical standards of the institutional and national research committee and with the 1964 Helsinki Declaration and its later amendments or comparable ethical standards. The Human Investigation Committee (IRB) of University Hospital Göttingen, application number: 3/12/17 approved this study. All procedures adhered to local and institutional laws and data protection regulations. Informed consent was not required as the study was retrospective in nature and involved the analysis of previously collected data.

## Results

**Fig. 1:**
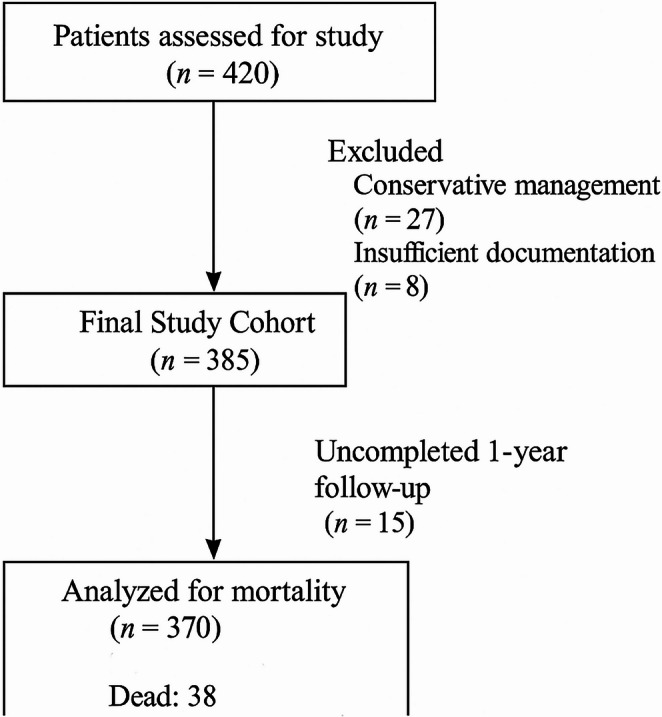
Patient flowchart showing study enrollment, exclusions, follow-up, and final mortality analysis

Three hundred and seventy of 385 patients were included in the study. Of these patients, 38 (10.2%) died within one year after surgery, of which 6 (1.6%) died within the first 30 days. Fifteen patients (3.9% of 385 patients) had incomplete 1-year follow-up and were therefore excluded from the primary mortality analysis (Fig. 1). Among all 370 patients, 41% had monosegmental involvement, 29% had two affected segments, 13% had three, 6% had four, and 11% had five or more segments. In contrast, the mortality cohort showed a higher proportion of patients with extensive disease (four segments: 16%; ≥5 segments: 21%). A chi-square test confirmed a statistically significant association between the extent of vertebral involvement and 1-year mortality (χ² = 13.83, *p* = 0.0079). The average follow-up period was 24 ± 3 months. The mean number of operated levels was 3 ± 2. We performed a subgroup analysis based on the duration of antibiotic therapy. Among the 370 patients in the cohort, 278 patients (75%) completed antibiotic treatment within 6–9 weeks, whereas 92 patients (25%) required a prolonged course of more than 9 weeks. Within the mortality cohort, 24 patients (63%) received antibiotics for 6–9 weeks, and 14 patients (37%) required treatment for more than 9 weeks. Statistical comparison between these subgroups did not reveal a significant difference in 1-year mortality (χ² = 2.58, *p* = 0.108). The most frequent causes of death within the one-year mortality cohort were multi-organ failure and fatal pulmonary embolism, each occurring in 10 patients (26%). This was followed by cardiopulmonary failure in 8 patients (21%), acute kidney failure in 6 patients (16%), and acute myocardial infarction in 4 patients (11%). It is important to note that many patients had multiple, overlapping conditions documented as contributing causes of death. A direct comparison of mortality rates between surgical procedures revealed a non-significant trend towards higher mortality in the 360° fusion group (16.7%, 8/48) compared to the dorsal instrumentation alone group (9.3%, 30/322); *p* = 0.13. The demographic, clinical, and surgical characteristics of the overall patient cohort and those who died within one year are summarized in Table 1.

**Table 1:** Characteristics and outcomes of mortality cohort (*n* = 38) vs. General surgical cohort (*n* = 370): A statistical comparison

Variable	Mortality Cohort (*n* = 38)	All Patients Cohort (*n* = 370)	*P*-Value
Demographics			
Male/Female (%)	60/40	57/43	0.69
Age (years, mean ± SD)	71.1 ± 12.0	69.2 ± 12.7	0.39
Diabetes Mellitus, n (%)	16 (42%)	115 (31%)	0.15
Cardiovascular Disease, n (%)	12 (32%)	102 (28%)	0.59
Chronic Kidney Disease, n (%)	10 (26%)	91 (25%)	0.82
Chronic Liver Disease	2 (5%)	15 (4%)	0.68
Lung Disease, n (%)	7 (18%)	47 (13%)	0.23
Current Smoker, n (%)	6 (16%)	28 (8%)	0.08
Drug Abuse, n (%)	4 (11%)	18 (5%)	0.11
Malignant Tumor, n (%)	4 (11%)	57 (15%)	0.4
Neurological Deficit, n (%)	20 (53%)	138 (37%)	0.05*
Sepsis, n (%)	7 (18%)	37 (10%)	0.16
Deformity, n (%)	8 (21%)	49 (13%)	0.21
Radiological Details			
Lumbar Spine, n (%)	22 (57%)	218 (59%)	0.87
Thoracic Spine, n (%)	15 (40%)	142 (38%)	0.89
Cervical Spine, n (%)	3 (8%)	25 (7%)	0.74
Spinal Epidural Abscess, n (%)	10 (26%)	110 (30%)	0.66
Spondylodiscitis Alone, n (%)	28 (74%)	260 (70%)	0.51
Surgical Details			
Dorsal Instrumentation Alone, n (%)	30 (79%)	322 (87%)	0.17
360° Fusion, n (%)	8 (21%)	48 (13%)	0.17
Surgery Duration (minutes, mean ± SD)	202 ± 75	200 ± 80	0.88
Robot-Assisted Surgeries, n (%)	17 (46%)	185 (50%)	0.6
Navigation System Surgeries, n (%)	16 (41%)	155 (42%)	0.95
Free- Hand Placement, n (%)	5 (13%)	30 (8%)	0.29
Wound Infection, n (%)	5 (13%)	50 (14%)	0.95
Microbiology			
Staphylococcus Aureus, n (%)	18 (48%)	185 (50%)	0.87
Staphylococcus Epidermidis, n (%)	3 (7%)	33 (9%)	0.78
Negative Cultures, n (%)	13 (34%)	107 (29%)	0.48
MRSA Infection, n (%)	4 (11%)	31 (8%)	0.66

### Univariate analysis

**Fig. 2:**
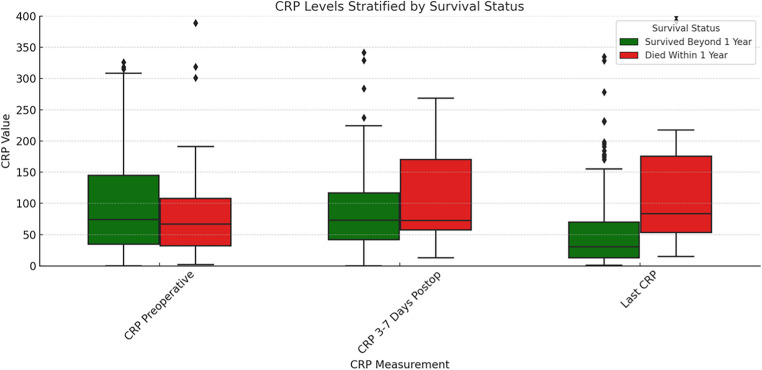
CRP levels at three time points (preoperative, postoperative, and last measurement) in survivors and mortality cohorts. The median last CRP level in the non-survivor group (100 mg/L) is significantly elevated compared to the survivor group (50 mg/L), indicating that higher CRP levels may correlate with a greater risk of mortality

**Fig. 3:**
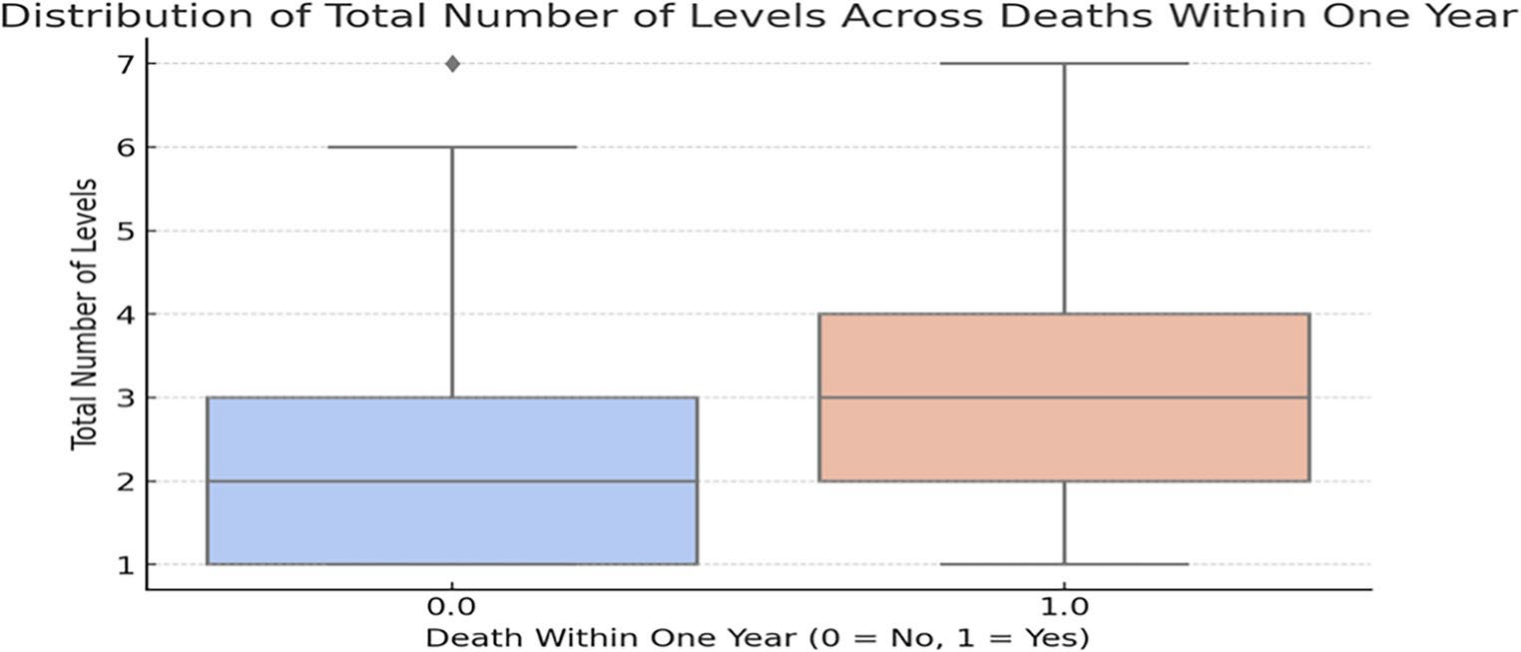
Distribution of the total operated levels in one year mortality. The median of the total operated levels in the non-survivor group (3 levels) is significantly elevated compared to the survivor group (2 levels), indicating that the total operated levels may correlate with a greater risk of mortality

Univariate analysis revealed several factors significantly associated with an increased risk of death. The last CRP levels (OR 1.009, 95% CI 1.005–1.014, *p* < 0.001) were found to be a strong factor associated with death (Fig. 2). The last recorded CRP measurement was obtained at a median of 4 months postoperatively [IQR: 2–6]. The total number of spinal levels treated surgically (OR 1.35, 95% CI 1.03–1.77, *p* = 0.0294) was also linked to mortality (Fig. 3). Additionally, the presence of neurological deficits (OR 2.82, 95% CI 1.21–6.55, *p* = 0.0158) was associated with increased mortality risk.

### Multivariate analysis

Multivariate analysis confirmed the last CRP (OR 1.01, 95% CI 1.004–1.016, *p* = 0.001) as an independent factor associated with mortality within one year of surgical treatment. While the odds ratio per 1 mg/L increase in last CRP was modest (OR = 1.01), this becomes clinically meaningful at higher thresholds. For instance, a 50 mg/L increase correlates with a 64% increase in odds of 1-year mortality, emphasizing the prognostic weight of persistently elevated inflammation markers. The number of operated spinal levels (OR 1.345, 95% CI 1.006–1.799, *p* = 0.046) was also an independent factor associated with mortality within one year of surgical treatment. On the other hand, neurological deficits did not retain statistical significance in the multivariate analysis (OR 1.676, 95% CI 0.621–4.522, *p* = 0.308), despite being significant in the univariate model. In a further analysis that re-included the patients that had incomplete 1-year follow-up (15 patients, 3.9% of 385 patients) under extreme-case assumptions (all alive vs. all dead at 1 year), the direction and statistical significance of the main predictors in the multivariate model (last CRP level and number of operated spinal levels) remained unchanged. These findings suggest that the small proportion of patients lost to follow-up is unlikely to have materially biased our estimates of 1-year mortality risk.

To address potential overfitting, we refitted the same model using penalized logistic regression (ridge). Effect sizes were directionally consistent and only moderately shrunk (Last CRP OR per SD = 2.12; operated levels OR per SD = 1.24), confirming the robustness of the primary model.

Assessment of calibration showed: Brier score = 0.091, indicating good overall accuracy. Hosmer–Lemeshow test: χ² = 4.27, df = 8, *p* = 0.83, showing no evidence of miscalibration. Calibration plot (deciles of predicted risk) demonstrated close agreement between predicted and observed 1-year mortality across the entire risk spectrum. All included variables had VIF values below 2.0, indicating no concerning collinearity and supporting their inclusion in the multivariate model.

### Discriminatory performance and threshold identification

Receiver operating characteristic (ROC) analysis of last CRP demonstrated good discriminatory ability for predicting 1-year mortality (AUC 0.78). The optimal data-driven threshold identified by Youden’s index was 47 mg/L (rounded from 47.1 mg/L to avoid artificial precision). At this cutoff, diagnostic performance was as follows: Sensitivity: 70.3% (95% CI 53.0–84.1), Specificity: 68.1% (95% CI 62.0–73.7), Positive predictive value (PPV): 23.9% (95% CI 16.2–33.0) and Negative predictive value (NPV): 94.1% (95% CI 89.8–97.0). Higher last CRP values were therefore strongly associated with increased 1-year mortality, while CRP ≤ 47 mg/L excluded late mortality with high negative predictive value.

**Fig. 4:**
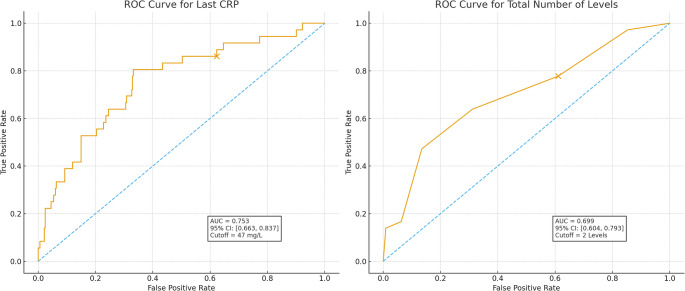
Receiver operating characteristic (ROC) curve for last CRP. The curve demonstrates the predictive performance of the last CRP measurement (in mg/L) for one-year mortality. The area under the curve (AUC) is 0.767 (95% CI: 0.682–0.852). The optimal cutoff value of 47 mg/L is indicated by the dot

For the number of operated levels, a cutoff of > 2 levels produced modest discriminatory performance (AUC = 0.638): Sensitivity: 54.1% (95% CI 36.9–70.5), Specificity: 56.2% (95% CI 49.9–62.3), PPV: 14.9% (95% CI 9.4–22.1) and NPV: 89.6% (95% CI 83.8–93.8). This threshold reflects the correlation between surgical extent and disease severity but demonstrates limited stand-alone predictive accuracy, consistent with its use as a clinical severity marker rather than a decision rule (Fig. 4).

Optimism correction and internal validation: Because both thresholds were derived from the present dataset, we assessed their robustness using bootstrap internal validation (500 resamples). The optimism in AUC was 0.018, yielding an optimism-corrected AUC of 0.76, only slightly lower than the apparent AUC (0.78) for last CRP. Similar negligible optimism was observed for the predictor “>2 levels.”These findings indicate minimal overfitting and reasonable internal validity, although the thresholds remain exploratory.

### Time-updated cox regression analysis

As a sensitivity analysis, we performed a time-to-event Cox proportional hazards model with CRP modeled as a time-updated covariate. Follow-up time was calculated from the date of surgery to death or 1 year, whichever occurred first. CRP values were treated as piecewise-constant, updated at each available measurement (preoperative, 3–7 days postoperatively, at discharge, and at last follow-up).

Higher CRP values over time were associated with an increased hazard of 1-year mortality (HR per 10 mg/L increase 1.03, 95% CI 1.00–1.05, *p* = 0.026), corresponding to an HR of 1.13 (95% CI 1.02–1.26) per 50 mg/L increase. In this model, the number of operated spinal levels was not significantly associated with mortality (HR per level 1.18, 95% CI 0.89–1.56, *p* = 0.25), whereas age remained independently associated with higher mortality (HR per 10 years 1.32, 95% CI 1.13–1.50, *p* < 0.001).

## Discussion

This study identifies clinically relevant predictors of long-term outcomes in a large cohort of patients with pyogenic spondylodiscitis. A key finding of this study is the identification of last CRP levels as a strong and independent factor associated with 1-year mortality in patients with pyogenic spondylodiscitis. Unlike preoperative or early postoperative CRP values, persistently elevated CRP levels—specifically those exceeding 47 mg/L—were significantly associated with poor long-term outcomes. This threshold demonstrated good discriminatory power and likely reflects ongoing or recurrent infection due to incomplete resolution or immune dysfunction. These results suggest that routine monitoring of CRP throughout the postoperative period, particularly beyond the immediate recovery phase, can provide critical prognostic information and may guide timely retreatment decisions.

In our cohort of 370 patients, the overall 1-year mortality rate was 10.2%, with an in-hospital mortality rate of 1.6%. These rates are consistent with those reported in the literature (approximately 8% at one year) and significantly lower than the mortality rates reported for patients managed conservatively, which range from 13% to 24% [9, 23, 26, 30]. These findings underscore the aggressive nature of spinal infections and highlight that early surgical intervention is associated with a markedly lower risk of both one year and in-hospital death [6, 14, 23, 25]. Preoperative and early postoperative CRP levels were not significantly associated with mortality, whereas elevated last CRP levels showed a strong correlation with 1-year mortality (Fig. 2). Although the last CRP was measured at a median of 4 months postoperatively [IQR: 2–6], this variability reflects individualized follow-up and recovery pacing. All CRP values used were recorded at the latest available timepoint prior to the 1-year mark and were treated as standalone factors associated with mortality, irrespective of exact timing. Sensitivity analyses were not performed to adjust for timing variability, which remains a limitation.

We routinely monitor CRP until the end of antibiotic therapy and only stop treatment once infection markers normalize. Imaging, primarily CT and MRI, complements CRP monitoring, though MRI accuracy can be limited by implant-related artifacts. CT helps assess implant and bone integrity but is not sufficient on its own to confirm the absence of infection [8, 16, 21]. Based on our results, we recommend checking infection markers at 3, 6 and 9 months after surgery. Our findings suggest that if CRP levels rise above 47 mg/L at any point, a more aggressive and immediate retreatment strategy should be considered, including appropriate antibiotics guided by blood culture results and established clinical protocols. Here, we should of course exclude other potential causes of elevated CRP levels, such as cancer, renal insufficiency, or clear infectious foci like urinary tract infections and pneumonia [20, 21]. The number of spinal levels requiring surgical intervention also emerged as a significant factor associated with one year mortality. This finding is consistent with the notion that more extensive disease requires a more complicated surgical approach, places a greater physiological burden on patients, and likely reflects a higher overall severity of infection. In fact, when more than two spinal levels were treated, the predictive capacity although modest relative to CRP (AUC = 0.638) remained clinically meaningful. However, in some cases, the extent of bone destruction and deformities may necessitate a more extensive approach, such as two stage surgery with 360° fusion [3, 17, 18, 27]. Vettivel et al. reported a one year mortality rate of 22.3% and found that increased age and the number of spinal levels involved elevated the one year mortality risk [26]. In our study, age was not identified as a risk factor, in contrast to the number of spinal levels operated on. The presence of neurological deficits significantly correlated with mortality in the univariate model, but it lost significance in the multivariate analysis. This may suggest that once surgical decompression and appropriate medical treatment are provided, neurological deficits per se do not independently drive patient survival if the underlying infection is adequately addressed. Aagaard et al., in a Danish nationwide cohort study, reported a one-year-mortality rate of 15%, primarily attributed to comorbidities, particularly substance abuse [1]. However, in our study, other factors such as age, preoperative comorbidities, epidural abscess, obesity, drug abuse, alcohol consumption, and the type of surgical treatment were not statistically significant, even though they may sometimes be associated with a more complex clinical course. It is also important to differentiate between in-hospital mortality and one year mortality, as they have different risk factors and mortality rates. Kehrer et al. reported a one year mortality rate of 20% after admission, linked to alcohol dependency, which decreased over time. However, short term mortality was associated with other risk factors such as infection, abscess formation, and neurological deficits [10]. Ziarko et al. and Akiyama et al. found that comorbidities such as heart failure, chronic kidney disease, and diabetes were strongly associated with increased in-hospital mortality. They also reported that infections with Pseudomonas aeruginosa carried the highest risk for in-hospital mortality [2, 30]. The timing of surgery is also critical. Neuhof et al. demonstrated that early surgical intervention significantly reduced the mortality rate to 4.2% within the first six weeks and shortened hospital stays compared to conservative management, which had a mortality rate of 24% [19]. This may explain our low 30 day mortality rate of 1.6%, as our patients with pyogenic spondylodiscitis received early surgical treatment. While we did not study the risk factors for one month mortality in our cohort, comparisons with the literature suggest that the risk factors for one month and one year mortality are distinctly different.

## Strengths and limitations

This study, which includes 370 patients, represents one of the largest cohorts analyzed in the context of pyogenic spondylodiscitis, strengthening the reliability and generalizability of the findings. However, several limitations should be acknowledged. The retrospective design may introduce inherent biases, including selection and information bias, which could affect the findings. In addition, postoperative CRP measurements after discharge were not obtained at standardized time points but were performed based on clinical indication. As a result, the time-dependent Cox analysis may be vulnerable to informative measurement bias and reverse causation (i.e., CRP may increase because patients are clinically deteriorating, rather than predicting deterioration). We therefore report the time-updated Cox model as an exploratory sensitivity analysis, whereas our primary conclusions are based on the simpler regression using the last available CRP value. Finally, because the proposed cutoffs were derived from internal data, their apparent predictive performance is likely optimistic. External validation in an independent cohort is required before these thresholds can be adopted as clinical decision points.

## Conclusion

Elevated CRP levels (> 47 mg/L) at a median of 4 months postoperatively [IQR: 2–6] and extensive spinal surgery involving more than two levels are strong, independent factors associated with one-year mortality in patients with pyogenic spondylodiscitis, after the exclusion of other potential causes for CRP elevation. These findings emphasize the critical importance of routine infection monitoring at 3, 6 and 9 months postoperatively to ensure timely intervention. Additionally, optimizing surgical strategies to balance efficacy and invasiveness, along with careful patient selection, is essential to improving outcomes.

## Data Availability

Anonymized data not published within this article will be made available by request from qualified investigators.
